# Kerstersia gyiorum Isolated and Identified From the External Auditory Meatus of an Immunocompromised Patient: A Case Report and Literature Review

**DOI:** 10.7759/cureus.76100

**Published:** 2024-12-20

**Authors:** Nasser F AlSunbul, Ali M Somily, Rima O AlOmar, Ahmed T AlHawamdeh, Nawaf Alqarni, Saad Aldraihem, Omar Alsulaiman, Abdullah K Alanzan

**Affiliations:** 1 Department of Pathology, College of Medicine, King Saud University, Riyadh, SAU; 2 Department of Pathology and Laboratory Medicine, College of Medicine, King Saud University, King Saud University Medical City, Riyadh, SAU

**Keywords:** alcaligenaceae, external auditory meatus, kerstersia gyiorum, matrix-assisted laser desorption ionization-time of flight (maldi-tof), microbiology culture

## Abstract

*Kerstersia gyiorum*, a rare Gram-negative pathogen first identified in 2003, belongs to the *Alcaligenaceae* family. Although infrequently reported, it has been isolated from various clinical infections, including wounds, and respiratory tract infections. Our case report highlights an unusual presentation of* K. gyiorum* in a 13-year-old girl with a complex medical history, associated with external ear pressure ulcers. The unusual, uncommonly identified organism was isolated from an ear swab and identified using MicroScan and matrix-assisted laser desorption ionization-time of flight mass spectrometry (MALDI-TOF MS). The bacterium exhibited sensitivity to multiple antibiotics but showed notable resistance to aminoglycosides and several common agents, emphasizing the importance of tailored therapy.

## Introduction

*Kerstersia gyiorum* is a very rare pathogen that belongs to the *Alcaligenaceaes* family. It was first reported in 2003 and was isolated as small (1-2 mm long) Gram-negative, coccoid cells that occur as single units [1,2,3]. It was initially observed on nutrient agar, where the colonies appeared flat or slightly convex with smooth edges, and their color ranged from white to light brown [3]. After it was first reported and although limited, further reports were conducted on isolating *K. gyiorum* from different infections [4]

*K. gyiorum* were reported from various wound infections including limb infections, e.g., lower-limb cellulitis from nonhealing, gradually enlarging, 10-cm ulcer of a 61-year-old obese patient on her left leg [5]; chronic leg ulcer from an 85-year-old with chronic venous insufficiency, with the organism isolated from a six-month-old wound measuring around 14 cm^2^ [6]; chronic osteomyelitis patient with a gangrenous wound [7]; respiratory tract infection isolated from bronchoalveolar lavage from a patient having a medical history of chronic respiratory failure requiring ventilator support and with reported bacteremia from a dialysis site infection [8]; and urinary tract infection of an 83-year-old lady with percutaneous nephrostomy [1].

The largest number of reports conducted about *K. gyiorum* came after infection of the diseased individuals having otitis media and isolated from purulent discharges of the ear [5,9-13].

## Case presentation

In this report, we present the case of a 13-year-old girl with a complex medical history, known to have cerebral palsy since birth, global developmental delay, blindness, and epilepsy that was controlled with Keppra 500 mg BID, in addition to having a gastrostomy (GGT) tube placed for feeding in January of 2023. Her guardian said she displayed symptoms of fever, diarrhea, and urine with an unpleasant odor, along with a reported increase in irritability and frequent moaning. The patient’s right ear showed signs of chronic perichondritis, likely due to prolonged pressure from lying on that side. The guardian reported these symptoms, in addition to ear discharge starting four weeks before hospital presentation.

They found that she was suffering from a case of gastroenteritis due to a viral infection, but her vital signs were stable and her baseline Glasgow Coma Scale (GCS) value was at 8/15. She had red and hot skin in the GGT site with the presence of an active discharge. Laboratory tests returned negative for common bacterial infections in the urine and stool where cultures were taken initially. Ear inspection while taking swab specimens showed a clear superficial wound on the outside.

The patient has previously and frequently been hospitalized and received numerous surgical interventions for her complex health needs. At the time of presentation, she is receiving supportive care including wound management and regular feeding through the GGT.

An ear swab from the external auditory meatus was received in the microbiology laboratory, a Gram stain was performed, pus cells were seen, and Gram-negative bacilli were identified. The sample was inoculated on solid media, such as a blood agar plate, chocolate, McConkey media, and thioglycolate liquid media. After 24 hours, a Gram-negative bacilli was isolated from solid media (colonial morphology). The identification and susceptibility were performed using the automated identification system MicroScan WalkAway Plus, Gram-Negative MIC50 panel (Beckman Coulter, CA, USA) and confirmed by matrix-assisted laser desorption ionization-time of flight (MALDI TOF) using VITEK MS MALDI TOF MS system (bioMérieux SA, La Balme les Grottes, France).

Additional antibiotics were tested using the E-test (Epsilometer, AB Biodisk, Solan, Sweden). The final identification of the organism was *K. gyiorum*. This bacteria was sensitive to piperacillin/tazobactam (Pip/Taz), ceftazidime, levofloxacin, meropenem, and imipenem and resistant to amikacin, gentamicin, tobramycin, ampicillin, azithormycin, cefazolin, cefourxime, cefoxitin, cefotaxime, ciprofloxacin, and trimethoprim/sulfamethoxazole (Table 1).

**Table 1:** Susceptibility testing profile of Kerstersia gyorium isolation using E-test

Antimicrobial agent	MIC (µg/mL)	Interpretation
Amikacin	32	Intermediate
Gentamicin	>8	Resistant
Tobramycin	>8	Resistant
Ampicillin	<=8	Resistant
Pip/Taz	<=16	Sensitive
Azithromycin	>16	Resistant
Cefazolin	>4	Resistant
Cefuroxime	>16	Resistant
Cefoxitin	>16	Resistant
Cefotaxime	>16	Resistant
Ceftazidime	4	Sensitive
Cefepime	16	Intermediate
Ciprofloxacin	2	Resistant
Levofloxacin	<=2	Sensitive
Imipenem	<=1	Sensitive
Meropenem	<=1	Sensitive
Trimethoprim / sulfamethoxazole		Resistant

She was started on intravenous fluconazole and ciprofloxacin, and with the addition of ofloxacin ear drops, the guardian was also advised to have water precautions to the ear.

## Discussion

This case is the first documented instance of *K. gyiorum* being isolated from a pressure ulcer in the external ear of an immunocompromised patient and to our knowledge the first reported in the Middle East and Saudi Arabia. Previous studies have reported this organism in cases of chronic otitis media, respiratory infections, and wound infections [3,5,8].

The organism seems to thrive in conditions of moisture, chronicity, and polymicrobial activity. This is consistent with the known propensity of this organism to colonize compromised tissue and in the current case, a pressure sore in a bedridden child would provide an obvious opportunity for exactly that. Although infections due to *K. gyiorum* have been reported most commonly in patients with otitis media, this case illustrates that the organism might cause a wider spectrum of involvement in soft tissue involvement especially the ear and auditory canal. It has a unique resistance profile, and due to its rarity, it is difficult for both identification and management. 

The use of advanced diagnostic methods, such as MALDI-TOF MS and MicroScan, were used for definitive identification in this case as demonstrated in other reports where MALDI TOF MS or similar techniques are revealed counter-indispensable to discriminate *K. gyiorum *from other pathogens mimicking this pathogen phenotypically [7,8].

The susceptibility of the isolate in this case was similar to that reported in other studies which showed sensitivity to piperacillin/tazobactam, imipenem, meropenem, and ceftazidime and resistance to aminoglycosides and some commonly used antibiotics such as ciprofloxacin [3,12].

## Conclusions

We presented a rarely occurring opportunistic pathogen,* K. gyiorum *in an immunocompromised patient that emphasizes that it is essential to recognize *K. gyiorum *at least in immunocompromised individuals. The accuracy of diagnostic strategies in such infections is paramount as seen from the successful isolation of this organism by the use of modern microbiological tools. Although uncommon,* K. gyiorum* exhibits a typical antibiogram profile that calls for patient-tailored antimicrobial treatment. In this instance, the organism's predilection for infecting compromised tissues in the host population is demonstrated by its finding in a chronic external ear pressure ulcer. The management of infections due to such rare pathogens requires early detection and a multidisciplinary therapeutic approach. Additional research is needed to define the clinical spectrum of *K. gyiorum* infection and ideal treatment regimens, as well as improve our understanding of preventative strategies for such organisms.
